# Comparative Analysis of Genipin-Crosslinked Carp Collagen Gels and Their Effects on L929 Fibroblast Growth and Differentiation

**DOI:** 10.3390/gels12030208

**Published:** 2026-03-02

**Authors:** Abdul Ghani, Yasuaki Takagi

**Affiliations:** 1 Graduate School of Fisheries Sciences, Hokkaido University, 3-1-1 Minato-cho, Hakodate 041-8611, Hokkaido, Japan; abdul.ghani.w3@elms.hokudai.ac.jp; 2 Faculty of Fisheries Sciences, Hokkaido University, 3-1-1 Minato-cho, Hakodate 041-8611, Hokkaido, Japan

**Keywords:** fish-derived collagen, genipin crosslinking, gel surface microstructure, L929 fibroblast, cellular responses, biomedical applications

## Abstract

Fish collagen exhibits lower denaturation temperatures and reduced mechanical stability than mammalian collagen, limiting its biomedical applicability and motivating the development of effective stabilization strategies. In this study, we address this challenge by establishing a simple and effective strategy to fabricate stable collagen gels from carp-derived collagen using phosphate buffer-induced gelation followed by genipin crosslinking. Gel properties were regulated by adjusting the gelation time prior to crosslinking. After 3 h of gelation followed by 24 h crosslinking, gels derived from carp skin, carp scale, and carp swim bladder exhibited relatively compact surfaces with irregular fibrils. Conversely, gels subjected to 24 h of gelation before crosslinking exhibited longer, thicker fibrils with less compact arrangement. Rheological and differential scanning calorimetry analyses showed that the crosslinked gels were thermally stable (40.84–47.17 °C) and structurally strong (G’: 11–50 kPa; G”: 1–8 kPa; tan δ: 0.05–0.17) gels. L929 fibroblasts cultured on these gels displayed distinct adhesion, spreading, and proliferation behaviors depending on gel microstructure. Furthermore, hyaluronic acid (HA) quantification showed that cells on the carp collagen gels stimulated HA production (25.05–26.15 ng/well). These results demonstrate that genipin-crosslinked carp collagen gels constitute tunable, cytocompatible collagen-based gels, with surface microstructure influencing fibroblast behavior, offering useful design insights for the development of stabilized fish-collagen materials for biomaterial applications.

## 1. Introduction

Fish collagen typically exhibits lower denaturation temperatures and mechanical stability than mammalian collagen, which limits its use in biomedical applications [1]. For example, a comparative thermal study revealed that fish skin collagen denatures at lower temperatures than bovine collagen, highlighting the need for strategies to stabilize its structure [2,3]. An effective strategy to reinforce fish collagen gels is crosslinking, which can enhance thermal stability but may also affect mechanical properties. Genipin, a crosslinker derived from the plant *Gardenia jasminoides*, has gained attention owing to its biocompatibility and ability to form stable covalent crosslinks within collagen networks [4]. For example, temperature-responsive gels composed of pepsin-solubilized collagen and genipin have been shown to gel rapidly under mild conditions and achieve tunable stiffness, making them suitable as injectable hydrogels [4]. Genipin crosslinking has been applied successfully in non-mammalian systems; for example, jellyfish collagen hydrogels treated with genipin exhibit significantly improved thermal stability, and embedded chondrocytes maintained metabolic activity over time [5]. Although traditional crosslinking methods (e.g., EDC/NHS) can also improve stability, the mild reaction conditions and favorable safety profile of genipin make it particularly suitable for tissue engineering [6].

The microstructure of collagen gels, including fibril diameter and alignment, can significantly influence cell behavior such as adhesion, spreading, proliferation, and extracellular matrix (ECM) production [7,8]. Collagen gels with different fibrillar architectures have been shown to alter cell spreading, focal adhesion development, and cell proliferation [9]. Furthermore, variations in matrix porosity and stiffness can modulate cell motility. In 3D collagen matrices, decreased porosity and stiffness restrict cell migration and alter cell morphology from dendritic to more flattened and polarized forms [10]. In fish collagen research, the collagen source plays a critical role in determining physicochemical properties and gel-forming behavior. A comparative study of type I collagen extracted from carp skin (CSK), scales (CSC), and swim bladders (CSB), and from sturgeon skin (SSK) and swim bladders (SSB) revealed pronounced differences in thermal stability, gel transparency, and fibrillar architecture [11]. Notably, carp-derived collagens exhibited higher thermal stability than sturgeon-derived collagens, with fibrillar structures evolving gradually over time. After 24 h of gelation, carp gels developed long and thin fibrils, whereas sturgeon gels formed shorter and thicker fibrils [11]. Such differences in fibril architecture are critical because they directly influence cell–matrix interactions. Beyond cellular morphological responses, cellular functions such as HA secretion are critically important. HA is a key ECM component that regulates cell motility, proliferation, and ECM-related activities [12]. HA is abundant in the ECM of the skin, which is the body’s largest organ, and plays a critical role in maintaining viscoelasticity, hydration, and rheological properties [13]. Cells cultured within or on collagen matrices often modulate HA production in response to the biophysical environment [14].

Our previous study [11] revealed the gel-forming ability of collagens obtained from different carp organs (skin, scales, and swim bladders), successfully fabricated collagen gels, and characterized their physicochemical properties. However, a key limitation identified was that these gels melted at 37 °C, preventing their use in cell culture experiments and limiting their applications. Thus, practical crosslinking to improve gel stability and denaturation temperature is required. Accordingly, the present study aims to develop genipin-crosslinked collagen gels prepared from carp skin, scales, and swim bladders, and to evaluate how crosslinking influences their microstructure, mechanical and thermal properties, and their ability to support cultured cells, i.e., the adhesion, spreading, proliferation, and hyaluronic acid secretion of L929 fibroblasts cultured on these gels.

## 2. Results and Discussion

### 2.1. Optical Images of Collagen Gels Crosslinked with Genipin

**Figure 1:**
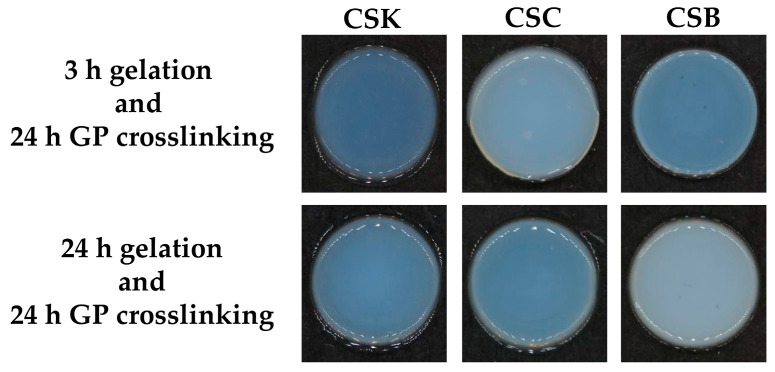
Optical images of 4% (*w*/*v*) collagen hydrogels derived from carp skin (CSK), scales (CSC), and swim bladder (CSB) after gelation for 3 or 24 h, followed by genipin (GP) crosslinking for 24 h at 20 °C.

Figure 1 shows the appearance of genipin-crosslinked collagen gels at two different gelation time points: 3 and 24 h. In both cases, the gels were crosslinked for 24 h after gelation. Following crosslinking, the gels prepared at both gelation times appeared completely turbid, suggesting a progressive crosslinking reaction and fibril formation within the collagen network. This observation indicates that genipin-induced crosslinking stabilizes the collagen fibrillar structure regardless of the initial gelation time, resulting in nearly identical macroscopic appearances. These findings align with those of previous studies reporting that collagen gel transparency is closely linked to the extent of fibril assembly [11].

### 2.2. Surface Microstructure of Genipin-Crosslinked Collagen Gel

Genipin has been widely studied as a biocompatible and efficient crosslinking agent for natural biomaterials, including collagen [15,16]. It specifically reacts with primary amine groups in collagen, initiating a ring-opening reaction via nucleophilic attack on the olefinic carbon atom of genipin. This leads to the formation of a covalent bond between genipin and the amino group of the polymer, generating an unstable intermediate aldehyde group. The intermediate is subsequently attacked by another amine group from a different polymer chain, forming a new covalent bond and ultimately yielding a stable crosslink [5].

**Figure 2:**
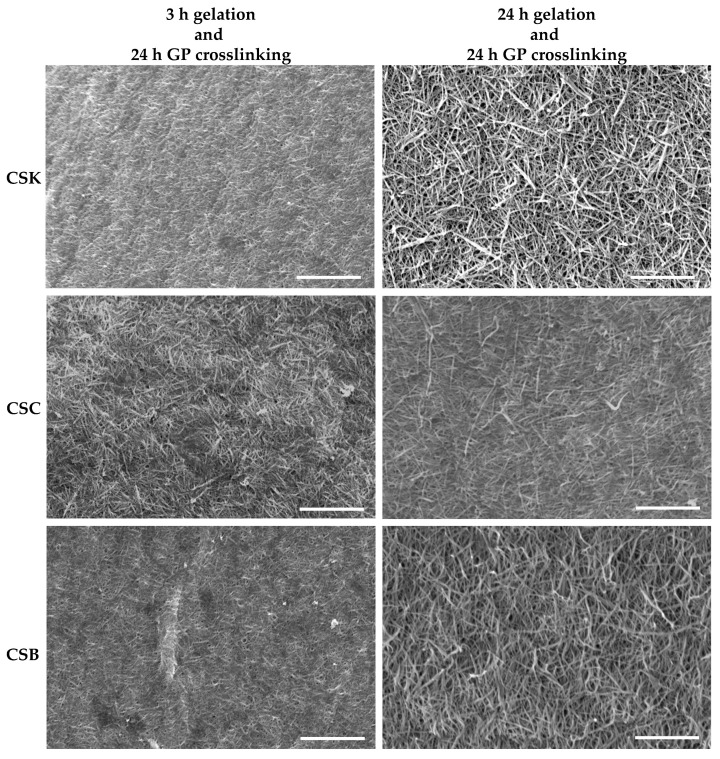
Surface SEM images of 4% (*w*/*v*) collagen hydrogels derived from carp skin (CSK), scales (CSC), and swim bladders (CSB) after gelation for 3 or 24 h, followed by genipin (GP) crosslinking for 24 h at 20 °C. Scale bars: 5 µm.

Figure 2 shows the surface microstructures of genipin-crosslinked collagen gels prepared from CSK, CSC, and CSB at two gelation times (3 and 24 h). In all conditions, the genipin crosslinking duration was fixed at 24 h. After 3 h of gelation followed by crosslinking, the CSK, CSC, and CSB gels exhibited relatively compact surfaces with irregular fibrillar networks made of short, thin fibrils. In contrast, gels subjected to 24 h of gelation before crosslinking showed more developed and less compact fibrillar networks across all collagen sources. These observations suggest efficient fibril maturation during prolonged gelation. Previous SEM analysis of non-crosslinked carp collagen gels showed similar trends [11]; however, after 3 h of gelation, they exhibited fewer fibrils with rougher surface microstructures, whereas after 24 h of gelation, the fibrils were well developed but more loosely packed, with larger interfibrillar spaces than in genipin-crosslinked gels. Therefore, gelation alone supports gradual fibril development and fusion, whereas subsequent genipin crosslinking more tightly and densely interconnects either the immature fibrillar network (3 h gelation) or the mature fibrils (24 h gelation). This indicates that genipin not only stabilizes the gelation-derived fibrils but also reorganizes and compacts the existing fibrillar network.

In jellyfish collagen, genipin treatment produced networks with fiber diameters of 0.58–0.66 µm, independent of genipin concentration [5]. Similarly, in tilapia collagen–alginate composites, genipin crosslinking reduced fibril bundle aggregation and produced a more compact network than EDC/NHS treatment, suggesting that genipin favors finer network organization [17]. These reports align with the present findings, in which genipin-crosslinked carp gels exhibited more compact fibrillar networks than non-crosslinked gels. Alternative approaches, such as using oxidized chondroitin sulfate (CSox) as a self-crosslinker, have shown that the microstructural features, including fibril thickness and network compactness, can be tuned by varying the crosslinker concentration [18]. Specifically, low CSox levels produced tighter fibril networks with thicker fibrils, whereas excessive CSox disrupted fibril self-assembly, leading to looser, bulk-like structures. In contrast, the present study indicates that genipin crosslinking applied for a fixed duration (24 h) influences fibril self-assembly and the organization of preformed fibrils; therefore, both gelation time and crosslinking play critical roles in defining the resulting collagen-gel microstructure. Collagen gelation in this study occurred through diffusion of phosphate buffer (pH 7.2) into the acidic collagen solution (pH 2.5). This diffusion-controlled process creates a moving gelation front in which collagen molecules progressively experience increased pH and ionic strength, promoting their self-assembly into protofibrils that laterally associate and elongate into mature fibrils. Consequently, fibrillogenesis is both time-dependent and spatially progressive. We hypothesize a mechanism that produces the microstructure of crosslinked gels as follows. After 3 h of gelation, the network consists of protofibrils with many exposed reactive amine groups that participate in genipin crosslinking. By 24 h, these protofibrils fuse into more mature fibrils, thereby reducing accessibility of reactive amine groups. Such a difference in fibrillar maturity directly affects genipin crosslinking. Genipin introduced after 3 h of gelation diffuses into a matrix rich in accessible amine groups, promoting extensive crosslinking and network compaction. In contrast, crosslinking after 24 h of gelation reorganizes fewer amine groups in mature fibrils, resulting in the final morphology closely related to that of fibrils before the crosslinking [9], with a weaker amount of network compaction compared with that in 3 h gelation gels. Thus, the surface microstructure of 24-h gelation gels shows more developed and less compact fibrillar networks.

### 2.3. Thermal Stability of Genipin-Crosslinked Collagen Gels

**Figure 3:**
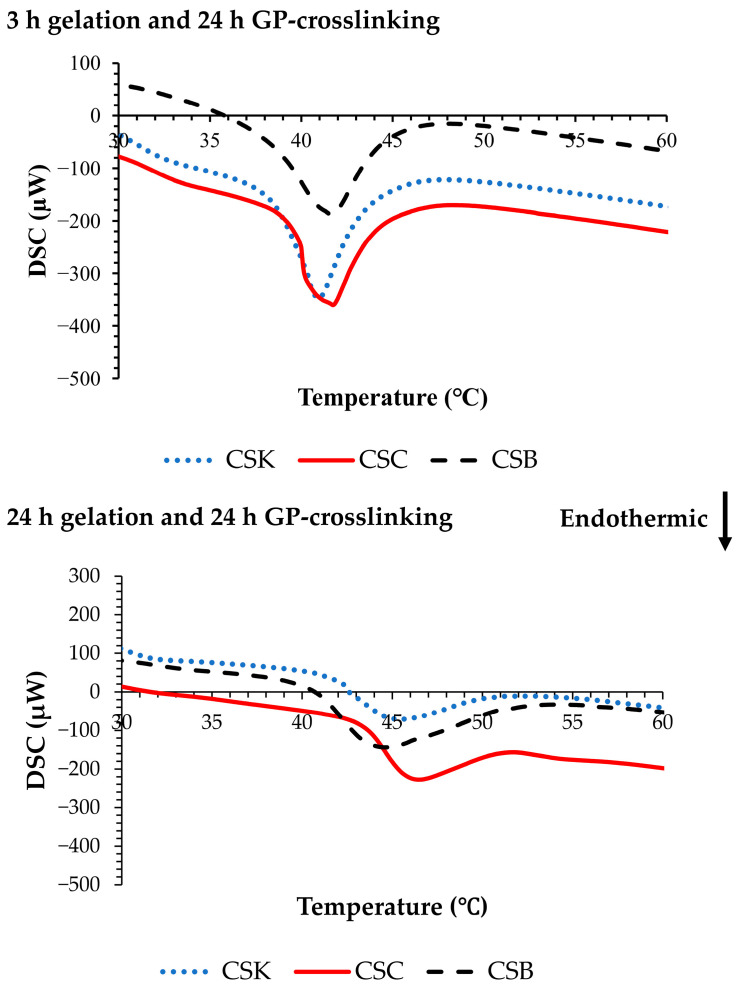
Differential scanning calorimetry (DSC) curves of 4% (*w*/*v*) collagen hydrogels derived from carp skin (CSK), scales (CSC), and swim bladders (CSB) after gelation for 3 or 24 h, followed by genipin (GP) crosslinking for 24 h at 20 °C.

Figure 3 shows representative differential scanning calorimetry (DSC) thermograms of the genipin-crosslinked collagen gels. In the 3 h gelation gels, the denaturation temperatures (Tds) were 40.84 ± 0.61 °C for CSK, 41.70 ± 0.80 °C for CSC, and 41.73 ± 0.70 °C for CSB (*n* = 3). Extending the gelation time to 24 h significantly increased Tds to 45.74 ± 0.77 °C for CSK, 47.17 ± 1.01 °C for CSC, and 44.30 ± 0.71 °C for CSB (*p* < 0.05). By comparison, non-crosslinked carp collagen gels showed lower Td values (36.79–39.19 °C) after 3 h of gelation, with modest increases upon extending gelation to 24 h [11]. This demonstrates that while gelation alone contributes to network stabilization, the incorporation of genipin significantly enhances thermal stability.

Other crosslinking approaches, including CSox, have shown clear increases in Td, from 60.8 °C in control samples to 68.3 °C at optimal crosslinker concentrations; however, excessive crosslinking reduced stability by interfering with fibril self-assembly [18]. Collectively, these findings emphasize that the denaturation behavior of collagen-based hydrogels is strongly modulated by various factors, such as the choice of crosslinker, crosslinking concentration, and gelation conditions. In this context, the present study confirms that genipin crosslinking substantially improves the stability of fish-derived collagen gels, increasing their denaturation temperature by approximately 3–5 °C relative to non-crosslinked gels [11].

### 2.4. Mechanical Properties of Genipin-Crosslinked Collagen Gel

**Figure 4:**
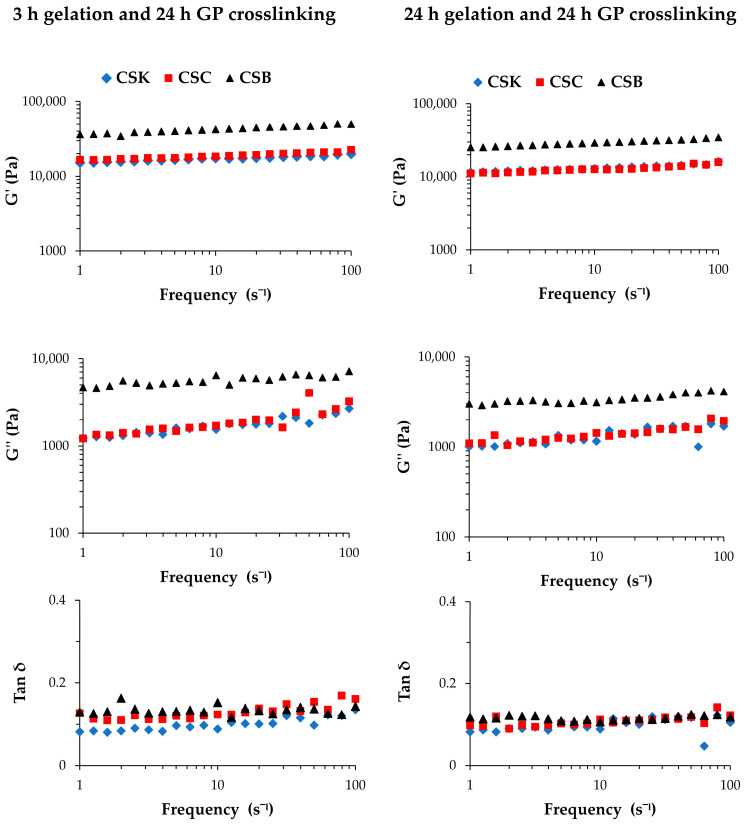
Storage modulus (G′), loss modulus (G″), and loss tangent (tan δ) of 4% (*w*/*v*) collagen hydrogels derived from carp skin (CSK), scales (CSC), and swim bladders (CSB) after gelation for 3 or 24 h, followed by genipin (GP) crosslinking for 24 h at 20 °C.

Figure 4 and Table 1 present the storage modulus (G′), loss modulus (G″), and loss tangent (tan δ) of genipin-crosslinked collagen gels derived from CSK, CSC, and CSB. In all samples, G′ was higher than G″ across the entire frequency range, confirming that the gels exhibited predominantly elastic behavior. The tan δ values remained below 0.2, indicating that the elastic response dominated over viscous dissipation and that genipin crosslinking effectively stabilized the collagen gel network. These results are consistent with previous studies reporting that collagen hydrogels typically exhibit G′ values substantially higher than G″, confirming their viscoelastic and predominantly elastic nature [18,19,20].

Rheological analysis revealed organ-dependent differences in the viscoelastic properties of the genipin-crosslinked collagen gels. After 3 h of gelation followed by crosslinking, CSB gels exhibited the highest G′ (34–50 kPa) and G″ (4–8 kPa), which were significantly greater than those of CSK (G′: 15–20 kPa and G″: 1–3 kPa) and CSC (G′: 16–23 kPa and G″: 1–5 kPa) (*p* < 0.05). At the same time, no significant difference was observed between CSK and CSC. A similar trend was observed for gels prepared with 24 h gelation. CSB showed the highest moduli (G′: 25–35 kPa and G″: 2–5 kPa), which were significantly higher than CSK (G′: 11–16 kPa and G″: 1–2 kPa) and CSC (G′: 11–16 kPa and G″: 1–3 kPa). Notably, for all collagen sources, gels crosslinked after 3 h of gelation showed higher G′ and G″ values than those crosslinked after 24 h, suggesting that a shorter gelation time allowed genipin to form more effective interfibrillar crosslinks, resulting in a denser and stiffer structure. These data well support our hypothesis that during the first 3 h gelation, many collagen molecules remain as protofibrils, promoting extensive crosslinking and network compaction by genipin. For the tan δ, CSK differed significantly from both CSC and CSB under both gelation conditions. These data indicate that CSK and CSC differ in their balance between viscosity and elasticity, although they exhibit similar stiffness (G′ and G″).

Genipin crosslinking markedly altered the viscoelastic behavior of carp collagen gels. Compared with non-crosslinked gels [11], crosslinked gels showed clear increases in both G′ and G″ across all organs, indicating network strengthening and stabilization through covalent crosslinks. Moreover, gels crosslinked after 3 h of gelation exhibited higher G′ and G” than those crosslinked after 24 h. Across crosslinked groups, CSB consistently showed the highest G’ and G″ values, followed by CSC and CSK. The tan δ values of crosslinked gels remained below 0.2 and were generally lower than those of non-crosslinked gels (0.2–0.4) [11], confirming that genipin treatment increased the elastic character of the gels. In non-crosslinked carp collagen gels, prolonged gelation produced organ-specific changes in viscoelasticity [11]. In particular, CSK gels exhibited a pronounced reduction in G″ accompanied by a marked decrease in tan δ, indicating reduced viscous dissipation and the formation of a more elastic, solid-like network. These effects were attributed to progressive fibril maturation and partial expulsion of bound water from the network during prolonged gelation. In the present study, genipin crosslinking enhanced the viscoelastic properties of carp collagen gels across all organ sources. The introduction of covalent crosslinks increased both G′ and G″ while maintaining low tan δ values, indicating improved mechanical stability and an elastic-dominant response.

Similar crosslinking-induced improvements in mechanical stability have been reported for collagen/CSox hydrogels, in which G′ and G″ increased and tan δ decreased relative to pure collagen gels, indicating enhanced elastic behavior associated with CSox crosslinking [18]. Riacci et al. [5] also reported that genipin-crosslinked collagen hydrogels maintain comparable G′ and G″ values over a range of genipin concentrations, underscoring the stabilizing role of genipin in preserving network integrity. Collectively, the present results confirm that genipin crosslinking significantly enhances the mechanical performance of collagen gels derived from different carp organs. The slightly higher moduli observed in samples crosslinked after 3 h of gelation further emphasize the importance of optimizing the gelation time before crosslinking to obtain stronger and more elastic collagen matrices.

### 2.5. L929 Cell Morphology on Genipin-Crosslinked Collagen Gel

**Figure 5:**
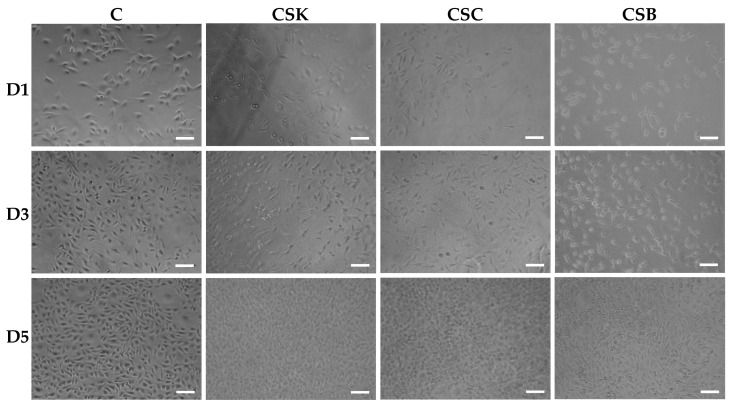
Phase-contrast microscopic images of L929 cells cultured for 1, 3, and 5 days on control wells (C) and collagen hydrogels (4% *w*/*v*) derived from carp skin (CSK), scales (CSC), and swim bladders (CSB). Gels were prepared by 3 h of gelation followed by 24 h of genipin (GP) crosslinking at 20 °C. Scale bars: 100 μm.

**Figure 6:**
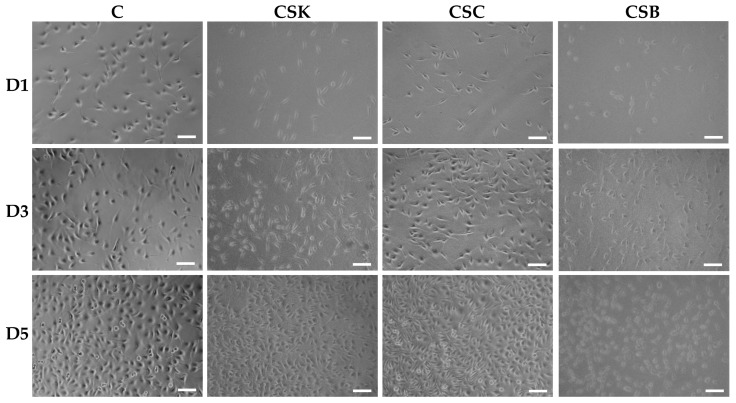
Phase-contrast microscopic images of L929 cells cultured for 1, 3, and 5 days on control wells and collagen hydrogels (4% *w*/*v*) derived from carp skin (CSK), scales (CSC), and swim bladders (CSB). Gels were prepared by 24 h of gelation followed by 24 h of genipin (GP) crosslinking at 20 °C. Scale bars: 100 μm.

**Figure 7:**
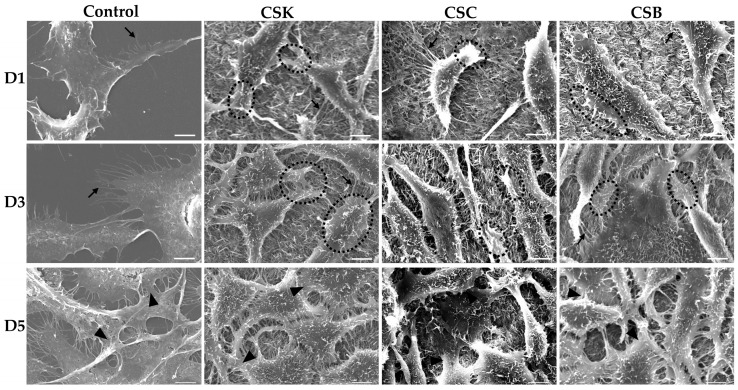
Scanning electron microscopy (SEM) images of L929 cells cultured for 1, 3, and 5 days on control wells and collagen hydrogels (4% *w*/*v*) derived from carp skin (CSK), scales (CSC), and swim bladders (CSB). Gels were prepared by 3 h of gelation followed by 24 h of genipin (GP) crosslinking at 20 °C. Scale bars: 5 µm. Arrows indicate filopodia extensions, circles show lamellipodia extensions, and arrowheads show intercellular contacts.

**Figure 8:**
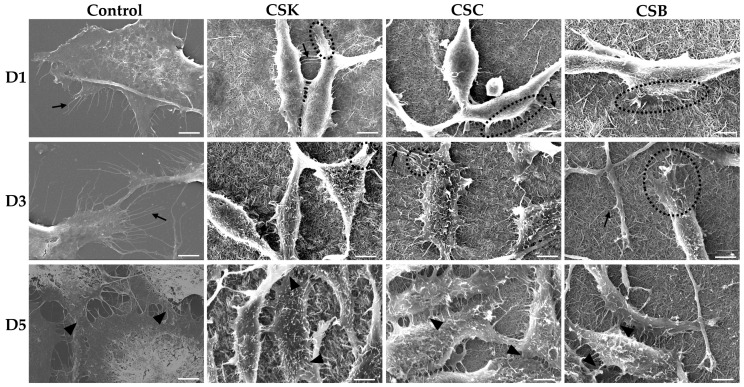
Scanning electron microscopy (SEM) images of L929 fibroblasts cultured for 1, 3, and 5 days on control wells and collagen hydrogels (4% *w*/*v*) derived from carp skin (CSK), scales (CSC), and swim bladders (CSB). Gels were prepared by 24 h of gelation followed by 24 h of genipin (GP) crosslinking at 20 °C. Scale bars: 5 µm. Arrows indicate filopodia extensions, circles show lamellipodia extensions, and arrowheads show intercellular contacts.

The phase-contrast and scanning electron microscopy images in Figure 5, Figure 6, Figure 7 and Figure 8 show the responses of L929 fibroblasts cultured on carp-derived collagen gels (CSK, CSC, and CSB) prepared with either 3 h of gelation (Figure 5 and Figure 7) or 24 h (Figure 6 and Figure 8), followed by 24 h of genipin crosslinking. Across all substrates, including the control cell culture, fibroblasts adhered rapidly and exhibited typical spindle-shaped morphologies as early as Day 1 (Figure 5 and Figure 6). However, cells on the control surface appeared noticeably flatter. With increasing culture time, both gelation conditions supported progressive cell proliferation, and morphological changes between Days 3 and 5 were more pronounced in the collagen gels than in the control cell culture wells (Figure 5 and Figure 6). No significant morphological differences were observed among the CSK, CSC, and CSB groups.

Overall, fibroblasts cultured on collagen gels prepared with either 3 h or 24 h gelation adopted more elongated, spindle-shaped morphologies than cells on control wells. Notably, cells cultured on the 24 h gelation gels exhibited a more pronounced spindle-like morphology and longer filopodial extensions than those on the 3 h gels. Such a finding suggests that more mature and less compact fibrils formed after 24 h of gelation provide a better supportive matrix for directional cell spreading. Conversely, cells on the 3 h gels appeared more uniformly extended in multiple directions, probably reflecting the influence of their denser and more compact fibrillar network.

SEM analysis (Figure 7 and Figure 8) further corroborated these observations. On Day 1, fibroblasts on the gels were predominantly in the initial attachment phase, exhibiting early spreading with filopodia- and lamellipodia-like extensions under both 3 and 24 h gelation conditions, suggesting active probing and anchoring to the collagen fibrils. These findings demonstrate successful initial adhesion and good surface biocompatibility of the gels for both gelation durations. By Day 3, the cells displayed greater spreading and a more elongated morphology, suggesting stronger substrate attachment. Increased cell density and closer proximity suggest active proliferation, accompanied by numerous intercellular contacts. Filopodia and lamellipodia were more abundant than on Day 1, reflecting enhanced matrix sensing and contact guidance. By Day 5, the collagen surfaces were extensively covered by dense fibroblast populations, with less distinct cell boundaries due to tight packing and intercellular networking. The mature, well-anchored morphology and robust intercellular connections observed on the gels indicate formation of a stable fibroblast layer with sustained viability, confirming favorable proliferation, adhesion, and cell-to-substrate interactions. No significant morphological differences were observed among cells cultured on the CSK, CSC, and CSB gels.

Compared with the collagen samples, cells on the control substrate remained more flattened and less spindle-shaped, and exhibited numerous thin filopodia (Figure 7 and Figure 8). By Day 5, they formed a dense, confluent monolayer primarily maintained through cell–cell contacts.

The surface topography of a scaffold strongly influences the behavior and interaction of cells with their environment [21]. During early cell–surface interactions, cells probe their environment using lamellipodia and filopodia, which are enriched in integrins [22,23,24,25]. During spreading, cells initially form nascent adhesions at their periphery [26], some of which mature into focal complexes [27]. Both nascent adhesions and focal complexes are unstable and can either disassemble within minutes or develop into stable focal adhesions [28]. Focal adhesions are multiprotein assemblies comprising integrins and associated proteins, including vinculin, talin, paxillin, tensin, zyxin, focal adhesion kinase (FAK), and α-actinin [29]; they anchor cells to the substrate and mediate mechanotransduction [30]. Recent evidence further demonstrates that fibrillar collagen substrates enhance the early development of focal adhesions, leading to activation and propagation of mechanotransduction signaling through FAK autophosphorylation and YAP1 nuclear translocation pathways, highlighting focal adhesions as essential mechanotransducers in fibroblasts cultured on fibrillar matrices [31]. In this study, fibroblasts on 3 h collagen gel surfaces exhibited a more spread, polygonal morphology with multiple outward protrusions, whereas cells on 24 h collagen gel surfaces were more elongated. Accordingly, a more mature, less compact fibrillar arrangement in the 24 h gels likely promotes formation of elongated focal adhesions and aligned stress fibers, thereby supporting cell elongation and contact-guided migration. In contrast, the densely packed fibrils in the 3 h gels may provide more frequent adhesion sites, facilitating multidirectional protrusions and a more isotropic organization.

The relationship between matrix architecture and fibroblast morphology observed in this study is consistent with previous reports demonstrating that substrate stiffness, fibril alignment, and surface topography strongly influence L929 cell spreading and filopodial behavior [32,33,34]. For example, Xu et al. [32] reported that collagen–silk fibroin membranes with supportive microfibrillar networks promoted abundant filopodia and elongated morphologies. Similarly, Meng et al. [34] showed that fibroblasts cultured on aligned sturgeon collagen fibrils developed increased cell heights and long, directional protrusions aligned with fibril orientation. The enhanced elongation observed on the 24 h gels in the present study is consistent with these findings and suggests that more developed fibrillar morphologies facilitate directional cytoskeletal organization. Shi et al. [35] using type II collagen fibrils from the sturgeon notochord reported that ATDC5 prechondrogenic cells initially exhibited shorter filopodia when cultured on fibril-coated surfaces than on molecule-coated surfaces. Over time, the number of filopodia did not increase markedly, but their lengths progressively increased. As the cell density increased, the cell bodies flattened, adopting rounded or oval shapes. By Day 14, the culture approached monolayer confluence, and filopodial extensions formed intercellular connections between adjacent cells.

In conclusion, these results indicate that genipin-crosslinked carp collagen gels effectively support L929 fibroblast adhesion, spreading, and proliferation on their surfaces. Gelation time modulates surface fibril organization: denser fibrillar surfaces in 3 h gelation gels promote multidirectional probing and uniform surface coverage, whereas more mature, loosely organized fibrils in 24 h gelation gels favor elongated cells with directional filopodia. Notably, no significant differences in cell morphology were observed among CSK, CSC, and CSB gels, indicating that the carp collagen source did not significantly affect L929 morphology under the conditions tested.

### 2.6. Cell Proliferation

**Figure 9:**
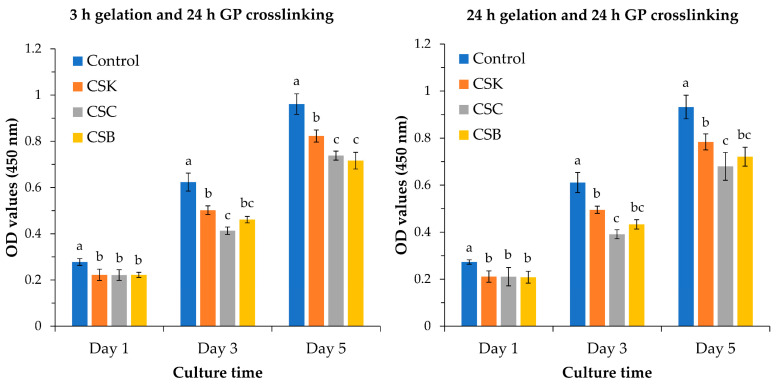
Proliferation of L929 cells cultured on control wells and collagen hydrogels (4% *w*/*v*) derived from carp skin (CSK), scales (CSC), and swim bladders (CSB). Gels were prepared under two gelation conditions: 3 h of gelation with 24 h of genipin (GP) crosslinking (**left**) and 24 h of gelation with 24 h of GP crosslinking (**right**). Data are shown as mean ± SD (*n* = 4). Different letters above the bars indicate statistically significant differences between groups at the same time point (*p* < 0.05).

The proliferation of L929 fibroblasts on collagen gels derived from CSK, CSC, and CSB was monitored over 5 d and compared with the tissue culture plastic control (Figure 9). Under both gelation conditions (3 and 24 h), the control group exhibited the highest proliferation across the culture period. On Day 1, all gel groups exhibited comparable values, with no significant differences among CSK, CSC, and CSB. By Day 3 and thereafter, significant differences were observed. Under both gelation conditions, CSK and CSB gels exhibited comparable cell proliferation, but only CSK showed significantly higher proliferation than CSC from Day 3 onward.

These proliferation outcomes likely reflect the microstructural features observed in this study: gels prepared with 3 h of gelation and 24 h of crosslinking formed compact fibrillar networks, whereas gels prepared with 24 h of gelation and 24 h of crosslinking produced less compact fibrillar surface structures. Previous studies support the role of the fibrillar architecture in regulating cell behavior. Meng et al. [34] reported slower proliferation of L929 cells on collagen fibril-coated substrates than on control surfaces and demonstrated that the fibril diameter modulates cell proliferation. They further reported higher proliferation on sturgeon skin collagen fibrils than on sturgeon swim bladder fibrils. Similarly, Moroi et al. [36] showed that fibrillar substrates consistently suppressed proliferation across multiple cell types (MC3T3-E1 pre-osteoblasts, ATDC5 pre-chondrocytes, 3T3-L1 pre-adipocytes, C2C12 pre-myocytes, and L929 fibroblasts), with coarse fibrils derived from sturgeon swim bladder collagen yielding the slowest growth. Shi et al. [35] corroborated this trend, reporting markedly slower ATDC5 growth on fibril-coated surfaces than on molecule-coated substrates using type II collagen from sturgeon notochord. Collectively, these findings indicate that the proliferation differences observed in our samples arise from differences in the underlying fibrillar network, which can regulate cell spreading and cell-cycle progression. In addition, several studies have shown that proliferation can be enhanced by modifying collagen with biologically active components, such as CSox [18].

### 2.7. HA Production

**Figure 10:**
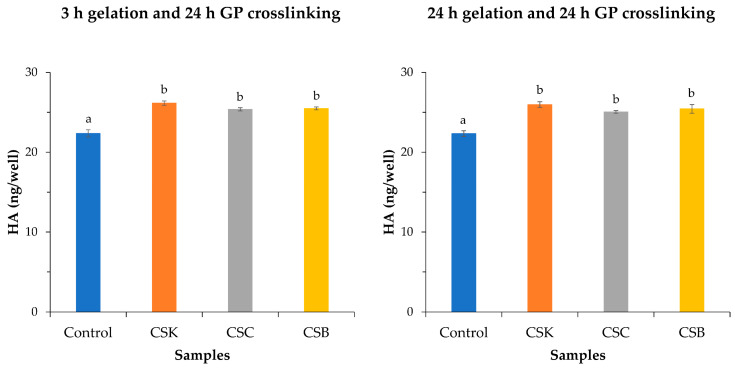
Hyaluronic acid (HA) secretion by L929 cells cultured for 3 days on control wells and carp-derived collagen hydrogels (4% *w*/*v*) derived from carp skin (CSK), scales (CSC), and swim bladders (CSB). Data are presented as mean ± SD (*n* = 3). Different letters indicate statistically significant differences among groups (*p* < 0.05).

Figure 10 shows HA production by L929 fibroblasts cultured on CSK, CSC, and CSB gels. Cells grown on CSK consistently produced the highest HA levels under both gelation conditions (3 and 24 h), reaching approximately 26.15 ng/well (3 h) and 25.96 ng/well (24 h), compared with 22.36 and 22.32 ng/well, respectively, on the plastic control. Although collagen from CSC and CSB also significantly increased HA secretion relative to the control, differences among collagen sources were not statistically significant. Gelation time did not significantly affect HA synthesis. Collectively, these findings indicate that carp-derived collagens enhance HA production by L929 fibroblasts compared with the plastic control.

The enhanced HA secretion observed on the carp-derived collagen gels aligns with the trends reported for other fibroblast systems that respond to ECM-like substrates. Sapudom et al. [37] demonstrated that both normal human dermal fibroblasts (NHFB) and cancer-associated fibroblasts (CAF) increased HA secretion when cultured within collagen-based matrices, with CAF producing substantially more HA than NHFB. Although these cell types differ from L929, the mechanistic principles may be comparable: collagen-rich 3D microenvironments can potentiate HA production, likely by stimulating adhesion-mediated signaling. Sapudom et al. [37] also reported that matrix-bound HA (500–1500 ng/mL/matrix) greatly exceeded HA detected in the supernatant (180 ng/mL per 10^4^ cells), indicating that fibroblasts preferentially retained HA within the matrix. The localized, punctate distribution of HA within collagen fibrils, as visualized by HABP staining, suggests that HA secretion occurs near focal adhesion sites [38], and HA may interact with concurrently deposited fibronectin [39,40]. Although the present study quantified only HA released into the supernatant, the elevated HA secretion by L929 cells on carp collagen gels suggests that a similar matrix-bound component likely exists and may be more substantial, as observed by Sapudom et al. [37].

Beyond matrix architecture, the biochemical composition of collagen scaffolds can modulate HA production. Liu and Sun [41] reported that fibroblasts cultured in a 3D gelatin-based matrix (3D-GF-PADM) secreted markedly more HA than cells in 2D cultures. They attributed this increase to amino acids such as proline and hydroxyproline—which are also abundant in fish-derived collagens—and to growth factors such as bFGF and PDGF-BB, which stimulate HA synthesis in fibroblasts [42] and can upregulate hyaluronan synthase activity [43].

The regulatory pathways governing HA biosynthesis further support the observed differences among scaffolds. Sapudom et al. [37] found that TGF-β1 enhanced HA production in NHFB and strongly increased matrix-bound HA in CAF, although earlier studies have reported variable dermal fibroblast responses to TGF-β1 [44,45,46]. These discrepancies underscore that the fibroblast phenotype and scaffold matrix used in culture experiments can affect cellular HA biosynthesis. In the present study, all carp collagen gels promoted HA secretion by L929 fibroblasts cultured on the collagen gel substrate relative to the control plastic well. These findings indicate that fibroblasts integrated both biochemical and structural cues from the collagen gel to regulate HA output. Collectively, these results show that carp-derived collagen gels provide a biologically active substrate that enhances fibroblast HA secretion, consistent with trends reported for other collagen-based biomaterial systems [47,48].

## 3. Conclusions

This study establishes a strategy for engineering functional collagen gels for two-dimensional cell culture on the gel surface using carp-derived collagen and genipin crosslinking via a simple fabrication method. By demonstrating that gelation time and genipin crosslinking modulate fibrillar architecture, this study demonstrates how processing parameters can tune collagen microstructure and, in turn, cellular responses. The improved thermal and mechanical stability conferred by genipin crosslinking further supports the use of these gels as mechanically robust biomaterials for surface-based applications, such as cell–material interaction studies. Importantly, L929 fibroblasts cultured on these gels exhibited substrate-dependent differences in adhesion, protrusive activity, and proliferation. Moreover, the ability of carp-derived collagens to enhance HA production underscores the biochemical functionality of these gels and their potential to support ECM-related activities. Overall, this study demonstrates that genipin-crosslinked carp collagen gels provide a tunable, biologically active surface that supports fibroblast adhesion, proliferation, and ECM-related activity and represent promising candidates for further investigation in 3D tissue-engineering applications.

## 4. Materials and Methods

### 4.1. Genipin-Crosslinked Collagen Gel Preparation

In this study, collagen gels were prepared from CSK, CSC, and CSB. The gels were fabricated using a previously reported method [11,49], with minor modifications. Briefly, 20 µL of collagen solution (4% *w/v*) in acidified deionized water (pH adjusted to 2.5 with HCl) was placed between two glass coverslips (24 × 50 mm and 24 × 24 mm) separated by 0.5-mm-thick silicone spacers. Gelation was initiated by introducing 0.1 M phosphate buffer (PB; pH 7.2) from the periphery of the collagen solution. The gelation front advanced inward by buffer diffusion, yielding a disk-shaped collagen gel (~6 mm in diameter and 0.5 mm thick). Two gelation conditions were employed: 3 and 24 h at 20 °C. After each gelation period, PB was replaced with 1 mM genipin (FUJIFILM Wako Pure Chemical Corp., Osaka, Japan) solution in 0.1 M PB (pH 7.2), and the gels were incubated for 24 h at 20 °C to induce crosslinking.

### 4.2. SEM of Genipin-Crosslinked Collagen Gels

The surface morphology of genipin-crosslinked collagen gels was analyzed using SEM (JSM6010LA, JEOL Ltd., Tokyo, Japan) following the sample preparation procedure described in [11]. Briefly, 0.5-mm-thick gels were fixed in 2.5% glutaraldehyde for 3 h at room temperature. The fixed samples were then rinsed with 0.1 M phosphate buffer (pH 7.2) for 3 h, with the buffer replaced every hour. Subsequently, the specimens were dehydrated through a graded ethanol series, followed by two immersions in t-butyl alcohol. The samples were freeze-dried from the t-butyl alcohol medium using a freeze dryer (FDU-2200, Tokyo Rikakikai Co., Ltd., Tokyo, Japan). Finally, the dried gels were sputter-coated with a gold–platinum using an auto fine coater (JFC-1600, JEOL Ltd., Tokyo, Japan) and examined under SEM.

### 4.3. Thermal Stability Assessment of Genipin-Crosslinked Collagen Gels

The thermal stability of genipin-crosslinked collagen gels was assessed by DSC (EXSTAR DSC6100, SII Nano Technology Inc., Chiba, Japan) according to the protocol described in [11]. Briefly, 0.5-mm-thick gels were prepared, and 15–20 mg of each specimen was accurately weighed and sealed in a 70 μL aluminum pan. DSC measurements were conducted over a temperature range of 30–60 °C at a heating rate of 2 °C/min, with a data acquisition interval of 0.2 s. A 0.1 M phosphate buffer (pH 7.2) was used as the reference. All analyses were performed under static air without a special gas atmosphere. The denaturation temperature (Td) was determined as the peak temperature corresponding to the maximum endothermic transition observed in the thermogram.

### 4.4. Mechanical Properties of Genipin-Crosslinked Collagen Gels

The mechanical properties of genipin-crosslinked collagen gels were evaluated using a rheometer (Ares-G2, TA Instruments, New Castle, DE, USA) following the testing conditions and frequency-sweep protocol described in [11]. Briefly, the 1-mm-thick gels were used for analysis. Dynamic frequency sweep measurements were performed over a frequency range of 1–100 s^−1^ at a constant shear strain of 0.2%. All tests were conducted using a parallel plate geometry at a controlled temperature of 20 °C.

### 4.5. L929 Fibroblast Cell Culture

L929 mouse fibroblasts (purchased from Cell Bank RIKEN BioResource Center, Ibaraki, Japan) were used to assess cell performance on the genipin-crosslinked collagen gels. Cells were maintained in minimum essential medium (MEM) supplemented with 5% fetal bovine serum (FBS) and 1% penicillin–streptomycin (PS) in a humidified incubator at 37 °C and 5% CO_2_, with medium being changed every 2 d. Cells at passages 24–28 were used for all experiments. Before cell seeding, the collagen gels were placed in a 96-well culture plate, rinsed twice with phosphate-buffered saline (PBS), and equilibrated in the culture medium for 30–60 min at 37 °C under 5% CO_2_. The medium was then removed, and L929 cells were seeded onto the gels and into control wells at a density of 3.0 × 10^3^ cells/well.

### 4.6. L929 Fibroblast Morphological Evaluation

The morphological responses of L929 cells cultured in control wells and on the genipin-crosslinked collagen gels were observed using a phase-contrast microscope (DMI6000BM; Leica, Wetzlar, Germany) and SEM (JSM6010LA, JEOL Ltd.). Phase-contrast images were acquired on Days 1, 3, and 5 after seeding. Samples were subsequently prepared for SEM, and morphological observations were conducted at the same time points to assess cell–surface interactions in detail. For SEM sample preparation, cells on both the gels and control wells were fixed and processed as described previously [11]. Before gold–platinum coating, the sidewalls of each control well were removed.

### 4.7. L929 Cell Viability and Proliferation Assay

L929 fibroblast viability and proliferation were evaluated using the CCK-8 assay kit (Dojindo, Laboratories Co., Ltd., Kumamoto, Japan) on Days 1, 3, and 5 of culture following the manufacturer’s instructions. Briefly, the medium was carefully removed, and the gels and control wells were washed three times with PBS. Fresh culture medium (180 µL) and CCK-8 reagent (20 µL) were then added to each well, followed by incubation at 37 °C for 1 h under 5% CO_2_. After incubation, 100 μL of the supernatant from each well was transferred to a new 96-well plate, and the optical density (OD) was measured at 450 nm using a microplate reader (Corona Electric Co., Ltd., Ibaraki, Japan).

### 4.8. Quantification of Hyaluronic Acid (HA) Secretion

HA secreted by L929 cells into the culture medium was quantified using a Hyaluronic Acid Quantification Kit (Cosmo Bio Co., Ltd., Tokyo, Japan), following the manufacturer’s instructions. Briefly, 100 µL of HA coating solution was added to each well of a 96-well plate, which was sealed and incubated at 26 °C for 1 h. Following incubation, the solution was discarded, and the wells were washed four times with 300 µL of 1× wash buffer using a multichannel pipette. After each wash, the plate was inverted and blotted on a paper towel to remove residual buffer. Subsequently, 200 µL of 1× blocking buffer was added to each well, and the plate was incubated at 26 °C for 30 min. The blocking solution was then removed, and the wells were washed once with 300 µL of 1× wash buffer. For sample loading, 50 µL of each HA standard solution and 50 µL of each sample were added to the appropriate wells, followed immediately by the addition of 50 µL of 1× biotinylated HA binding protein (Biotin-HABP) to all wells. The plate was sealed, gently mixed for 30 s, and incubated at 26 °C for 1 h. The solution was then discarded and the wells were washed four times with 300 µL of 1× wash buffer. Subsequently, 100 µL of 1× HRP-avidin was added to each well and incubated at 26 °C for 1 h, followed by four washes with 300 µL of 1× wash buffer. Next, 100 µL of substrate solution was added to each well, and the plate was incubated in the dark for 20–30 min for color development. The reaction was stopped by adding 100 µL of stop solution to each well and OD was measured immediately at 450 nm using a microplate reader. Results are reported as nanograms of HA per well (ng/well) in the culture supernatant collected after 3 d of culture.

### 4.9. Statistical Analysis

All quantitative experiments were performed in triplicate, and the results are presented as mean ± standard deviation (SD). Statistical analyses were performed using one-way analysis of variance (ANOVA) followed by Tukey’s HSD post hoc test. Differences between groups were considered statistically significant at *p* < 0.05.

## Figures and Tables

**Table 1:** Storage modulus (G′), loss modulus (G″), and loss tangent (tan δ) of 4% (*w*/*v*) collagen hydrogels derived from carp skin (CSK), scales (CSC), and swim bladders (CSB) after gelation for 3 or 24 h, followed by genipin (GP) crosslinking for 24 h at 20 °C, measured over a frequency range of 1–100 s^−1^.

3 h gelation and 24 genipin-crosslinking			
Samples	G′ (kPa)	G″ (kPa)	tan δ
CSK	15–20	1–3	0.08–0.14
CSC	16–23	1–5	0.11–0.17
CSB	34–50	4–8	0.12–0.16
24 h gelation and 24 genipin-crosslinking			
Samples	G′ (kPa)	G″ (kPa)	tan δ
CSK	11–16	1–2	0.05–0.12
CSC	11–16	1–3	0.09–0.14
CSB	25–35	2–5	0.11–0.12

## Data Availability

The data supporting the findings of this study are included in the article, and further inquiries can be directed to the corresponding author.
